# Process of developing the National Health in All Policies Framework to address social determinants of health in Tanzania Mainland: lessons learned, 2018 - 2022

**DOI:** 10.11604/pamj.supp.2023.45.1.39693

**Published:** 2023-06-08

**Authors:** Edward Mbanga, Catherine Joachim, Omar Ilyas, Raynold John, Leticia Rweyemamu, Sally Mtenga, Grace Mhalu, Alphoncina Nanai, Neema Kileo, Mary Kessi, Nemes Iriya, Maximillian Mapunda, Peter Phori, Doris Kirigia, Nicole Valentine, Michaela Told, Abigail Marwa, Zabulon Yoti

**Affiliations:** 1 Ministry of Health, Tanzania Mainland, Dodoma, Tanzania,; 2 Prime Minister’s Office, Dodoma, Tanzania,; 3 World Health Organization, Country Office, Dar es Salaam, Tanzania,; 4 Ifakara Health Institute, Dar es Salaam, Tanzania,; 5 World Health Organization, Regional Office for Africa, Brazaville, Congo,; 6 World Health Organization, Headquarters, Geneva, Switzerland

**Keywords:** Health, social determinants, policy framework, multi-sectoral collaboration

## Abstract

**Introduction:**

social determinants of health (SDH) are the non-medical factors that contribute to various infectious and non-infectious diseases in Tanzania. Studies suggest that SDH account for 30-55% of health outcomes globally. Most SDH are outside the mandate of the health sector; hence, multi-sectoral collaboration through Health in All Policies (HiAP) is critical. Health in All Policies looks at public policies across sectors that consider health implications of decisions, seek synergies, use resources and avoid harmful health impacts to improve population health and health equity. This paper demonstrates lessons learned from the process of developing National HiAP Framework in Tanzania Mainland to address SDH. It is expected that countries will be able to learn and adopt what deems fit in their context as they address SDH to improve population health.

**Methods:**

different methods were used to promote multi-sectoral collaboration in addressing SDH through HiAP. They included consultations with Prime Minister’s Office (PMO) as the coordinator of Government business for their buy-in. High-level advocacy meetings of Directors of Policy and Planning and Permanent Secretaries from sectoral ministries were conducted to move forward the HiAP agenda. Capacity building was provided for sectoral Ministries to understand HiAP concept and SDH. Interministerial collaboration meetings were convened to bring sectors together to identify SDH issues and key areas for inter-sectoral collaboration and develop National HiAP Framework to address SDH. Health in All Policies Secretariat coordinates the HiAP activities.

**Results:**

it has been noted that almost every sectoral ministry has a health component in its policy which contributes to the Tanzanian population’s health. In this regard, every sectoral ministry has a role to play in addressing SDH for sustainable development. Political will is key in moving forward the HiAP agenda. The role of PMO is significant to ensure inter-sectoral collaboration. Achieving the national and Sustainable Development Goals require strong collaboration among sectors and stakeholder coordination at all levels through HiAP.

**Conclusion:**

implementing HiAP is a win-win situation. It enhances inter-sectoral collaboration, benefiting each sector to achieve its health-related strategic indicators and ultimately achieve national and global goals.

## Introduction

The social determinants of health are the non-medical factors that influence health outcomes. These are the conditions in which people are born, grow, work, live and age and the wider environment shapes the conditions [1]. Some determinants include income and social protection, education, unemployment and job insecurity, working conditions, food insecurity, housing, basic amenities and the environment, early childhood development, and access to affordable health services. Most of these determinants are mainly outside the direct scope and mandate of the health sector; hence, the health sector alone cannot address them. Studies suggest that social determinants of health account for 30-55% of health outcomes [1]. Addressing social determinants of health is fundamental for improving health and reducing health inequities.

Evidence shows that the social determinants of health contribute to various infectious and non-infectious diseases in Tanzania and other settings. These determinants include education, norms, gender inequities, income and wealth, and distribution. Others include early childhood care, lifestyles, working conditions, job security, food security and social safety nets. Moreover, housing, including access to safe water and sanitation, weak health systems such as human resources, governance, health-seeking behavior, poor transportation networks, climate change and environmental threats are among them [2-8]. The Tanzania Demographic and Health Survey, 2015/16 revealed that non-communicable diseases account for 27% of total deaths exerting more pressure at the family and national level both economically and socially, leading to poverty and a reduction in productivity level [9]. The situation necessitates more public awareness of non-communicable disease risk factors, including physical activity, healthy diet and health-seeking behavior, to which non-health sectors contribute the majority. Besides the nutritional considerations, the agricultural chain has several determinants of health. About 65% of Tanzanians are dependent on agriculture [10]. Investing in agriculture guarantees success in health. The Sustainable Development Goals (SDGs), 2016-2030, emphasize multi-sectoral actions whereby all 16 goals contribute to health. This means that population health is not influenced solely by the Ministry of Health’s efforts but rather by a wider variety of stakeholders and policies.

In this context, an inter-sectoral collaboration thematic discussion group on social determinants of health (SDH) was formed by the health sector-wide approach (SWAp) partners in December 2017. The aim was to accelerate progress in achieving the objectives of the Health Sector Strategic Plan IV (HSSP IV), 2015-2020 [11]. It was agreed that the Ministry of Health and other sectoral ministries would thoroughly review related key sectors’ policies. The objective was to identify existing opportunities to help formulate a joint plan and common result accountability framework toward a realistic inter-sectoral collaboration [12]. Following that, the World Health Organization (WHO) supported the Government in the efforts to address SDH through a Health in All Policies (HiAP) approach to achieving the objectives of HSSP IV and the subsequent Health Sector Strategic Plan V, 2021-2026 [13]. Health in All Policies is an approach to public policies across sectors that considers the health implications of decisions, seeks synergies, uses resources and avoids harmful health impacts to improve population health and health equity [12].

In Tanzania and elsewhere, there are multiple health policy guidelines, but there is limited evidence to demonstrate the process of developing those guidelines. As such, the country’s specific best practices are rarely documented and learned. This paper, therefore, aims to describe the lessons learned and the process of developing the National Health in All Policies Framework in Tanzania Mainland.

## Methods

We used the following methods in the formulation of HiAP in Tanzania **Mainland:** 1) joint annual health sector review; 2) consultation and advocacy meeting; 3) desk review; 4) joint planning and accountability framework; 5) high-level meeting; 6) capacity building; 7) working session; 8) refresher training; 9) review meetings; 10) Health in All Policies Secretariat.

**Joint annual health sector review:** it was agreed by the SWAp partners during the 18th Joint Annual Health Sector Review (JAHSR) in December 2017 in Dar es Salaam to form an inter-sectoral collaboration thematic discussion group to address social determinants of health (SDH). The group comprised the Ministry of Health, Ministry of Water, Ministry of Education, Science and Technology, Ministry of Works, Transport and Communication, Ministry of Agriculture, President’s Office of Regional Administration and Local Government, and President’s Office Public Service Management and Good Governance. The group developed policy recommendations to address SDH, which were concluded in the Annual Policy Commitments of 2018/2019 during the JAHSR Policy meeting on 24th January 2018 in Dar es Salaam.

**Consultation and advocacy meeting:** the consultation and advocacy meetings on the HiAP concept to address SDH were conducted at Prime Minister’s Office (PMO) as the coordinator of sectoral ministries for their buy-in. The HiAP concept was well received by the PMO, which led to the planning of interministerial collaboration meetings by the PMO and the Ministry of Health (MoH). This was done by MoH in collaboration with WHO in April 2018 in Dar es Salaam.

**Desk review:** as a first-phase Interministerial collaboration meeting, we conducted the desk review of sectoral ministries’ policies from 7th - 12th May 2018 in Morogoro. This was done under the coordination of PMO in collaboration with MoH. Thirteen sectoral ministries participated. Two resource persons represented each sectoral ministry: the Director of Policy and Planning and the Policy Analyst to strengthen inter-sectoral collaboration. The team analyzed health-related areas from the sectoral policies and identified areas for inter-sectoral collaboration.

**Joint planning and accountability framework:** the second phase interministerial collaboration meeting was conducted from 21st - 25th May 2018 in Morogoro for joint planning to address social determinants of health. During the meeting, the 13 sectoral ministries developed a Draft National HiAP Plan of Action, July 2018/19 and proposed a HiAP Governance structure to strengthen inter-sectoral collaboration. Health in All Policies Roadmap and Accountability Framework were also developed.

**High-level meeting:** the high-level meeting of Permanent Secretaries (PSs) from sectoral Ministries was organized by the Directors of Policy and Planning (DPPs), who met in Dar es Salaam from 28th - 30th September 2018. This was done under the coordination of PMO to prepare all relevant meeting materials for PSs’ advocacy and their buy-in. The actual PSs meeting took place on 1st October 2018 in Dar es Salaam where 14 sectoral ministries participated. From the meeting, the PSs agreed on: HiAP ownership, the appointment of a sectoral Ministries Liaison person, capacity building of HiAP technical team (i.e., DPPs, Liaison person on minimum), finalization and operationalization of Joint Plan of Action (POA), linkages with existing health Sector Wide Approach (SWAp) reviews, and submission of HiAP Framework at high-level Ministerial Policy Forums.

**Capacity building:** each sectoral ministry appointed the HiAP focal point for HiAP activities. HiAP capacity building workshop to address social determinants of health (SDH) was conducted for Directors and Assistant Directors of Policy and Planning and Policy Analysts from 15 sectoral ministries from 17th - 20th September 2019 in Dar es Salaam. The training was conducted in collaboration with WHO Regional Office for Africa. The training focused on the global perspective of the HiAP and its concepts, the social determinants of health and countries’ experiences in inter-sectoral actions. Sectoral ministries shared their experiences on existing multi-sectoral forums in the country, including nutrition, road safety, water, One Health and School Water, Sanitation and Hygiene (WASH). They developed activities and indicators for HiAP implementation. The team agreed on a working session to finalize the draft National HiAP Action Plan, Governance structure and collaboration matrix.

**Working session:** we conducted the Policy Analysts´ working session to finalize HiAP framework from 7th - 11th June 2021 in Morogoro. Fourteen sectoral ministries shared the progress of activities implemented in the past two years (2018/19 - 2019/20) to allow quick synergy in finalizing the HiAP Framework. We reviewed HiAP Framework, identified SDH priority areas and led ministries for HiAP coordination. It was proposed that the PMO organize another capacity-building session to assist in finalizing the HiAP Framework as some of the sectoral ministries’ staff were new due to job transfers within the Government. It was recommended to involve more sectors in the HiAP initiative. The activity was also delayed due to the COVID-19 pandemic.

**Refresher training:** we organized HiAP refresher training for focal points, programs, academia and NSAs to allow them to understand the HiAP concepts and SDH and be able to finalize the draft National HiAP Framework. The training took place from 8th - 12th November 2021 in Morogoro. Seventeen sectoral ministries participated, including those from Zanzibar. Academia and NSAs also participated in learning and sharing their experiences for improving the HiAP Framework. International consultant was engaged during the training to help participants finalize the Framework. The team reviewed the HiAP Framework and provided their input. They also provided inputs to the draft WHO Guidance on Sustainable Multi-sectoral Collaboration in addressing social determinants of health through HiAP. Tanzania was selected as a pilot country. The draft guidance was used to shape the draft National HiAP Framework. A national consultant was engaged to collect inputs for the guidance. The international consultant was tasked to share the consolidated draft HiAP Framework for sectoral Ministries to share it with their stakeholders for input. It was agreed that the consultant would produce the advance draft by February 2022.

**Review meetings:** a Directors of Policy and Planning (DPPs) meeting was conducted in Arusha from 27th - 29th July 2022 to review the draft National HiAP Framework for addressing SDH in Tanzania Mainland. DPPs from 18 sectoral Ministries participated in the review of the HiAP Framework. The review started with an orientation on HiAP concepts and SDH, as some of the DPPs were new. The HiAP Framework was intensively reviewed by the DPPs, who provided their inputs, including a suggestion to develop an SDH monitoring and evaluation plan as a component of the HiAP Framework for measuring the progress of the indicators from the National Five-Year Development Plan. Engagement of all sectoral ministries in the HiAP initiative was proposed. HiAP meeting to review SDH monitoring and evaluation plan as a component of the HiAP Framework was held from 1th - 2th September 2022 in Morogoro. Sixteen sectoral Ministries participated, which were represented by Policy Analysts. Sectoral ministries’ SDH monitoring and evaluation plans were presented and reviewed, and comments were provided accordingly. Prime Minister’s Office was advised to follow up with the missing sectoral Ministries for submission of their SDH monitoring and evaluation plans.

**Health in All Policies Secretariat:** we organized the HiAP Secretariat meeting from the 13th - 17th December 2022 in Morogoro. The HiAP Secretariat comprised PMO, MoH and President’s Office Regional Administration and Local Government (PORALG). Ifakara Health Institute, Mzumbe University, and WHO were invited to support the Government. The Secretariat was divided into four groups to finalize the draft National HiAP Framework and prepare for a high-level meeting of Permanent Secretaries (PSs). The Secretariat incorporated DPPs inputs into the Framework, reviewed sectoral ministries’ monitoring and evaluation plans and prepared a presentation and speech for the PSs meeting. Gaps were identified in sectors’ monitoring and evaluation indicators, where some were not adequately defined. Prime Minister’s Office circulated the HiAP Framework to DPPs for their review and improvement (specifically sectors M&E indicators matrix) and briefing to their PSs before the high-level meeting.

## Results

We found several key issues that are critical for the development of the National HiAP Framework for addressing SDH. Amongst the critical ones is political will. As the HiAP involves a whole of Government approach that is, sectoral Ministries and Agencies, it needs blessings from the high-level officials of the Government. Coordination of the sectoral Ministries is another factor where the role of the Prime Minister´s Office as the coordinator of Government business in this regard, is inevitable. We learned the importance of Interministerial collaboration to address SDH and health inequities in a holistic manner. This is a win-win situation as sectors do not only achieve their health-related goals but also individual sectoral goals and ultimately national and global goals. These findings are demonstrated in Table 1 of the lessons learned from the process of developing the National Health in All Policies Framework to address social determinants of health in Tanzania’s Mainland.

**Table 1:** lessons learned from the process of developing the National Health in All Policies Framework to address social determinants of health in Tanzania Mainland

Issue	Lesson learned
Political will	There is a political will to address determinants of health through HiAP. This was evidenced by high-level advocacy meetings with PMO and PSs and their commitment to own HiAP by appointing focal points from their offices to participate in HiAP activities.
Governance mechanism and the role of the Prime Minister’s Office (PMO)	PMO coordinates most of the multi-sectoral work in Tanzania since one of its main roles is policy and coordination of Government business. Not less than six multi-sectoral collaboration mechanisms are under PMO, including Nutrition, Violence Against Women and Children, HIV, Disaster Management, One Health and HiAP. All these mechanisms address SDH. Regarding HiAP, PMO currently coordinates 25 sectoral ministries in collaboration with the lead sectoral ministry, the Ministry of Health, through the whole of Government approach. The Governance mechanism and PMO’s role are key in strengthening Inter-ministerial collaboration for population health and equity.
The role of sectoral ministries in addressing social determinants of health (SDH)	Every sectoral ministry has a role to play in addressing SDH as sectors' policies can potentially affect health and cause inequities in health. Using HiAP approach addresses policies such as those influencing water, transport, housing and urban planning, environment, education, agriculture, finance, trade, and economic development so that they promote health, well-being and health equity. Such development should not be addressed in silos. Achievement of the seventeen SDGs requires strong collaboration among sectors and stakeholder coordination at all levels.
The role of the Health in All Policies (HiAP) focal points	Each sectoral ministry appointed the HiAP focal point for HiAP activities whom WHO trained on HiAP and SDH. The training and experience sharing on existing multi-sectoral collaboration have built capacity and commitment for HiAP across sectoral ministries. These can facilitate ownership and contribute to smooth planning and coordination of health in all policies activities.
Multi-sectoral collaboration can strengthen response in health emergencies	The impact of the COVID-19 pandemic highlighted the link between health, well-being, social protection and the economy, demonstrating that a multi-sectoral collaborative approach is essential in a crisis.

We also found out that each sectoral Ministry has a health-related component in its policy which demonstrates that each sector has a role to play in the population’s health as their policies and actions have an impact on health and equity. This is further illustrated in Table 2 of the analysis of health-related areas from policy documents from a few selected sector Ministries, SDG goals they address and areas for inter-sectoral collaboration. The identified priority areas for inter-sectoral collaboration will strengthen the Interministerial collaboration in addressing the social determinants of health and ultimately benefit each sector in achieving individual and national goals.

**Table 2:** analysis of health-related areas from policy documents from a few selected sector Ministries and areas for inter-sectoral collaboration

Ministry	Policy/strategy	Sustainable development goal	Health determinant issue	Area for inter-sectoral collaboration
Vice President’s Office	National Environmental Policy 2021	13 climate action, 15 Life on land	Food security and poverty reduction	Environmental conservation
Prime Minister’s Office		1-17 coordination	Policy harmonization Health in all Policies	Further strengthening of Inter-ministerial collaboration
Prime Minister’s Office	National Action Plan for Health Security (NAPHS)		Plan to reduce morbidity, mortality, disability and socio-economic disruptions	Strengthening and sustaining the capacity to prevent outbreaks and other health emergencies
Ministry of Labour and Employment	National Employment Policy 2008	8 good jobs and economic growth	Poverty reduction of the people	Improvement in health status of the people
Ministry of Finance and Planning	FYDP III 2021/22 - 2025/26	1-17 facilitation	Allocation of financial resources	Improvement of flow of funds to related sectors
Ministry of Health	National Health Policy, 2007	3 good health	Mandated with health status of the nation and technical in the implementation of health in all policies	Include all ministries to achieve Health in all Policies
	Health Sector Strategic Plan 2021-2026			
	National Strategy for Accelerating Sanitation and Hygiene for All 2020 -2025		Elimination of open defecation, achieve universal access to improved sanitation	
Water and Irrigation	National Water Policy, 2002	6 clean water and sanitation	Vision 2025 increasing water access to 95% urban and 85% rural.	Ensure access to safe water by extending water to all health facilities, all regions to have laboratory for testing water safety
President's Office, Regional Administration and Local Government Tanzania (PORALG)		1-17 implementation	Proper implementation of Joint Health Facilities Rehabilitation, Primary Health Services Development Program	Strengthen Health Governance (Decentralized SWAp)
Ministry of Agriculture	National Agriculture Policy - 2013	2 no hunger, 15 life on land	Increased and sustainable productivity, production of food and non-food agricultural commodities, reduction in the prevalence of under-nutrition and malnutrition	Food security to the population

We noted that when developing a policy document, it is important to ensure its alignment with the national priorities to be able to measure progress in its implementation in line with the national goals which also facilitates the achievement of the global goals. As most of the SDH is measured under the National Five-Year Development Plan (FYDP), it encourages sectors to value the importance of inter-sectoral collaboration in achieving national goals. Table 3 shows the indicators matrix in the National HiAP Strategic Framework for addressing SDH. The indicators were selected from the third National Five-Year Development Plan (FYDP III) by sectoral Ministries to monitor HiAP priority interventions for addressing SDH and improving population health and equity. The HiAP Framework will be presented in 2023 at high-level meetings of Permanent Secretaries and Ministers for endorsement and implementation at national and sub-national levels.

**Table 3:** indicators matrix in the National HiAP Strategic Framework for addressing SDH in Tanzania Mainland: indicators selected from FYDP III

Indicator	Baseline 2019/20	Target 2025/26
**Human development - poverty**		
Human development index	0.57	0.60
Proportion of the population below the basic need poverty line (national)	26,4%	22%
Income inequality (national)	0.38	0.36
**Health**		
Infant mortality rate per 1,000 births	36	30
Under five mortality rate per 1,000 births	50	40
Births attended by a skilled health worker (%)	80	85
Maternal mortality ratio per 100,000	220	180
Life expectancy (years)	66	68
National HIV prevalence rate (%)	4.7	3.1
Health expenditure, public (% of Govt. expenditure)	10	12.2
**Food security and nutrition**		
Prevalence of stunting in children aged 0 - 59 months	31.8	24
Prevalence of wasting (weight for height) among children 0-59 months	3.5	Less than 5
Prevalence of low birth weight (LBW) at birth	6.3	Less than 3
Proportion of women aged 15-49 years with anaemia	44	22
Prevalence of global acute malnutrition among children 0-59 months	3.5	Less than 5
Prevalence of overweight among children 0 - 59 months	2.8	Less than 5
Overweight among adults aged 15-49 years	30	Less than 29
Proportion of households accessing adequately iodised salt	62	80
Rate of exclusive breast feeding (EBF)	59	65
**Agriculture**		
Volume of total horticultural production per year (tons)	6,556,102	14,600,000
Food sufficiency ratio (%)	124	130
**Livestock**		
Livestock mortality rate	27	12
Livestock vaccination rate	25	50
Milk production (billion litres)	3	4.3
**Fisheries**		
Per capita consumption fish (KG)	8.5	10.5
Contribution to the National Animal Protein intake	30	35
**Education**		
% of primary schools with clean water	49.3%	60%
Adult literacy rate	77.6%	81.6%
**Water**		
Percentage of rural population with access to piped or protected water as their main source	70	85
Percentage of households in rural areas with improved sanitation facilities	36	75
Percentage of regional centres population with access to piped or protected water as their main source	84	95
Percentage of households connected to convention public sewer systems in regional centres	13	30
Percentage of Dar es salaam population with access to piped or protected water as their main source	70	85
Percentage of household connected to convention public sewer systems in Dar es Salaam	13	30
**Urbanisation, housing**		
Number of residential licences issued to property owners in unplanned settlements	1,738	20,000
Proportions of villages with land use plans	19.3	52.7
Number of towns with up-to-date general planning schemes (master plans)	24	54
**Social protection**		
Coverage of health insurance scheme (%)	50	80
Coverage of the social security scheme (%)	7.3	13.1
Proportion of children with disability attending primary school (%)	80	100
Number of destitute elders with health insurances cards for free medical services	166,866	500,000
Percentage of children in beneficiary households aged 0-24 months old attending health facilities regularly	94	95
**Security**		
Rate (%) reduction in crime rates in the country	5,2	35
Rate (%) reduction in road accidents	30.5	35
**Environmental protection**		
Percentage share of GDP from sustainable utilisation of forest, water and marine resources	4	7
Increased consumption of alternative charcoal in urban areas (Tons)	100	100,000
Loss of biodiversity reduced (%)	30	20
Environmental pollution reduced (%)	-	5

## Discussion

This paper presents lessons learned and processes from developing the National Health in All Policies Framework in Tanzania Mainland from 2018 - 2022. From the processes, we have learned the following:

**The political will to address determinants of health through health in all policies:** this is evidenced during consultation and advocacy meetings with the Prime Minister´s Office and later high-level meetings of PSs where they committed to own HiAP by appointing focal points from their offices to participate in HiAP activities and strengthening the Interministerial collaboration.

**Governance mechanism and the role of Prime Minister’s Office:** the MoH does not have the mandate to call sector Ministries together. Prime Minister’s Office coordinates most of the multi-sectoral work in Tanzania since one of its main roles is policy coordination. From this, we learned that the role of the PMO in policy and coordination in collaboration with the Ministry of Health is significant to ensuring inter-sectoral collaboration for improved health outcomes. This has helped strengthen inter-ministerial collaboration amongst sector ministries in HiAP. The interministerial collaboration meetings led to identifying the health determinant issues, areas for intersectoral collaboration and developing the draft National Health in All Policies Framework to address the health determinants. A monitoring and evaluation indicators matrix was developed in line with FYDP III to monitor the achievement of the priority areas for inter-sectoral collaboration.

**The role of sectoral ministries in addressing social determinants of health:** we learned that almost every sectoral ministry has a health component in its policy that contributes to the Tanzanian population’s health. In this regard, every sectoral ministry has a role to play in addressing SDH for sustainable development. Government sectors’ policies can potentially affect health and inequities in health. Using a health-in-all-policies approach addresses policies such as those influencing water, transport, housing and urban planning [14-16]. Other policies are those on the environment, education, agriculture, finance, trade and economic development [17-20]. The aim is for the policies to promote health, well-being and health equity [21]. This recognition has raised awareness that development should not be addressed in silos. Achieving the 17 SDGs requires strong collaboration among sectors and stakeholder coordination at all levels.

**The role of the Health in All Policies focal points:** we have also learned that commitment of the HiAP focal points from the sectoral Ministries facilitates ownership and contributes to smooth planning and coordination of health in all policies activities. The appointment of the HiAP focal points, training in HiAP and SDH and sharing of experiences on existing multi-sectoral collaboration mechanisms in the country has built capacity and commitment for HiAP across sectoral ministries.

**Multi-sectoral collaboration can strengthen response in health emergencies:** we realized the impact of the COVID-19 pandemic, which highlighted the link between health, well-being, social protection, and the economy, demonstrating that a multi-sectoral collaborative approach is essential in a crisis. The pandemic has also revealed the challenges and opportunities for implementing the Health in All Policies approach during and after the pandemic [22]. We have noted several limitations during the HiAP Framework development processes. The COVID-19 pandemic delayed the HiAP initiative processes in 2020 due to the ban on physical meetings and other Government competing priorities. Staff turnover and new appointments within the Government also delayed the finalization processes of the HiAP Framework. Refresher training was undertaken for new HiAP focal points to understand the HiAP concepts and be able to finalize the Framework.

## Conclusion

We conclude that all sectors must understand that implementing HiAP is a win-win situation and that HiAP enhances inter-sectoral collaboration, benefit each sector to achieve its health-related strategic indicators and ultimately achieve national and global goals. We recommend that all relevant sectors and policies should take health issues into account and work jointly through health in all policies to overcome the social, economic and environmental determinants of health to improve the health and well-being of Tanzanians.

### What is known about this topic

- Multi-sectoral collaboration is crucial in addressing the social determinants of health;
- Health in All Policies is an approach that can be used to bring the different sectors and actors together in a whole of Government and whole of society approach to address the health determinants comprehensively for the healthier population;
- It helps in achieving not only the SDG 3 but also the other sustainable development goals as it is a win-win approach where sectors complement each other to achieve their individual sector goals and at the same time contributes to the achievement of the SDG 3.

### What this study adds

- The paper demonstrates the processes and lessons learned in the formulation of the national HiAP framework for addressing SDH in Tanzania;
- The methods and processes used helped in wider engagement of all Government sector Ministries, political will and Interministerial collaboration to address determinants of health;
- Documentation of the methods, processes and lessons learned would help countries that will undergo similar process to follow.
